# Diagnostic accuracy of percutaneous transthoracic needle biopsy among peripheral pulmonary lesions: a multicenter observational study

**DOI:** 10.1097/MS9.0000000000002539

**Published:** 2024-09-10

**Authors:** Reza Basiri, Farzad Sharifnezhad, Amir H. Jafarian, Sara Samadi, Amirreza Zarghi

**Affiliations:** a Lung Disease Research Center, Faculty of Medicine Mashhad University of Medicine Sciences; b Department of Internal Medicine, Faculty of Medicine, Mashhad University of Medical Sciences; c Cancer Molecular Pathology Research Center, Mashhad University of Medical Sciences; d School of Medicine, Mashhad University of Medical Sciences, Mashhad, Iran

**Keywords:** CT scan, percutaneous transthoracic needle biopsy, pulmonary lesion

## Abstract

**Introduction:**

The diagnosis of peripheral pulmonary lesions (PPL) poses a significant challenge, prompting the widespread utilization of various modalities to ensure the precision in diagnosis. This study aims to assess the diagnostic accuracy of computed tomography-guided percutaneous transthoracic needle biopsy (CT-PTNB) in the context of pulmonary malignancy.

**Methods and materials:**

This multicenter retrospective observational study, included 1317 cases of CT-PTNB performed on adult patients with PPLs from January 2018 to December 2022 in Mashhad, Iran. The pathology results of CT-PTNB from 94 cases were compared to the definitive pathology results obtained through methods such as surgery to assess the sensitivity, specificity, and overall accuracy of CT-PTNB in diagnosing of pulmonary malignancy.

**Results:**

CT-PTNB exhibits an accuracy of 82.98%, with sensitivity and specificity rates of 75.41 and 91.43%, respectively. This study underscores the issue of false-negative results in CT-PTNB and underscores the importance of integrating clinical, radiological, and additional diagnostic modality to guide diagnostic decisions.

**Conclusion:**

In this large-scale multicenter study, the accuracy of CT-PTNB for diagnosis of pulmonary malignancy is acceptable but fairly low compared to previous studies.

## Introduction

Highlights

- CT-PTNB exhibits an accuracy of 82.98%, with sensitivity and specificity rates of 75.41 and 91.43%, respectively.
- This study underscores the importance of integrating clinical, radiological, and additional diagnostic modality to guide diagnostic decisions.
- In this large-scale multicenter study, the accuracy of CT-PTNB for diagnosis of pulmonary malignancy is acceptable but fairly low compared to the previous studies.

Peripheral pulmonary lesions (PPLs) refer to pulmonary lesions of the subsegmental bronchi^1^. There is complexity in the detection of PPLs via the conventional transbronchial biopsy (TBB) due to their extraluminal location. Therefore, alternative methodologies have recently replaced conventional TBB. As an alternative, endobronchial ultrasound-guided TBB, despite its advancement, still often fails to diagnose PPLs in 51% of cases^2,3^. Conversely, image guided percutaneous transthoracic needle biopsy (PTNB) emerges as a highly accurate method for diagnosis of PPLs. Different imaging modalities like ultrasound, computed tomography (CT), and electromagnetic navigation have been introduced as the guidance for conducting PTNB^4^.

According to the previous guidelines and studies, computed tomography-guided percutaneous transthoracic needle biopsy (CT-PTNB) is preferred modality due to its superior spatial resolution^5,6^. This minimally invasive procedure is a commonly utilized to provide a tissue for various thoracic lesions, particularly PPLs larger than 10 mm^7^. Despite the high diagnostic accuracy of CT-PTNB at 90–99%^8^, it is significantly associated with complications like hemoptysis and pneumothorax^9^, with notably higher rates compared to TBB. Hence, TBB may be the favored choice for not only centrally located lesions but also for PPLs when a positive bronchus sign is observed^10^.

Owing to the relative advantages and disadvantages of CT-PTNB, it still remains uncertain whether this approach is optimal for diagnosing PPLs. On the other hand, the accuracy of diagnostic modalities is highly dependent on the operator’s expertise and the quality of equipment and technology used, potentially leading to variations in diagnostic accuracy across different locations. To the best of our knowledge, it is little known about the utilization of CT-PTNB in Iran. Accordingly, the present study aimed to investigate the diagnostic accuracy of CT-PTNB for lung cancer in a large study of Iranian patients.

## Methods and materials

### Patients

This was an observational multicenter study at two university hospitals in Mashhad, Iran, from January 2018 to December 2022. All adult patients (≥18 years) with a peripheral lung lesions larger than 10 mm in chest computed tomography scan (CT scan) and available CT-PTNB pathology results included. Individuals who had pathology results of surgery or video-assisted thoracoscopic surgery (VATS) were included to determine the diagnostic accuracy of CT-PTNB.

### Procedure

All CT-PTNBs were performed by one of the three interventional radiologists under commercially unenhanced CT scan in the prone, supine or lateral decubitus position depending on the location of the nodule. When a definitive diagnosis was not attainable or the diagnosis remained uncertain and ambiguous, the patient was a candidate for open lung biopsies or VATS to establish a diagnosis. All biopsy samples, CT-PTNB or surgery, were examined by three pathologists.

### Definition of outcome

Based on the methods of the previous studies, the results of CT-PTNB were categorized into five groups: specific malignancy, atypical cells or suspicious malignancy, nondiagnostic results, nonspecific benign disease, and specific benign diseases^11,12^. The ‘specific benign disease’ encompassed in this study comprises benign lung tumors, infectious pneumonia, pulmonary tuberculosis (TB), silicosis, vasculitis, and several other conditions. The term ‘nonspecific benign disease’ encompasses several pathological conditions, including acute or chronic nonspecific inflammation, granuloma formation, localized fibrosis, or specimens that exhibit no malignancy^13^. ‘Nondiagnostic results’ were operationally defined as a pathological report of CT-PTNB specimens that just exhibited the presence of blood, necrosis, normal lung parenchyma, or an inadequate amount of tissue to establish any definitive diagnosis.

The definite diagnosis was based on the pathology results obtained from surgery or VATS, and these results were compared to the pathology findings from CT-PTNB in order to assess the diagnostic accuracy of CT-PTNB. To facilitate this comparison, all pathology results were categorized as positive if they indicated evidence of a specific malignancy, atypical cells, or suspicion of malignancy. Conversely, results were classified as negative if they indicated the absence of malignancies. Furthermore, instances where the original pathologic reports showed that the specimen was inadequate or insufficient for an accurate pathological diagnosis were also included as cases with nonevaluable results due to insufficient specimens^8,14^.

### Statistical analysis

Categorical variables were reported as proportions (%) and continuous variables as means with SD. The previous study suggests that when evaluating the accuracy of a diagnostic test, utilizing a ‘3×2 table’ approach known as the intention to diagnose approach is more effective than the traditional ‘2×2 table’ in terms of transparent reporting and managing nonevaluable results^14^.

Therefore, a 3×2 table was utilized, in which the results of CT-PTNB were compared to the definitive pathology results obtained from surgery or VATS. These results were further categorized to derive the following five categories: true positives, false positives, false negatives, true negatives, and nonevaluable results.

Based on the intention-to-diagnose principle, nonevaluable findings caused by insufficient specimens were accounted for as false negatives in sensitivity calculations and false positives in specificity calculations^14^. SPSS version 16 was used for the statistical analysis.

## Results

**Figure 1:**
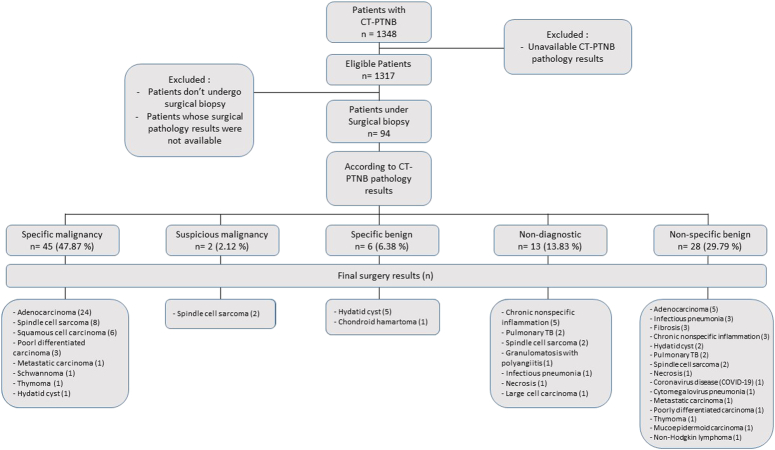
Flowchart of the study.

Overall, 1348 CT-PTNB cases were identified over a 4-year study period, of which 31 cases were excluded due to unavailable CT-PTNB pathology results. Out of 1317 remaining cases, only 94 cases had definite diagnosis pathology available and included to determine the CT-PTNB accuracy analysis (Fig. 1).

The research encompassed a study of 1317 individuals, with a mean age of 61.23 (SD=15.04) years with 57% being male (*n*=761). The specific malignancy was the most common CT-PTNB finding with 57.93%, followed by nonspecific benign results (20.43%), nondiagnostic results (11.77%), specific benign results (7.89%), and suspicious malignancy (1.97%) (Table 1).

**Table 1:** The mean age and sex distribution of study participants based on CT-PTNB findings (*n*=1317)

		Sex	
	Mean age (SD)	Male (%)	Female (%)
Specific malignancy *N*=763 (57.93%)	62.15 (14.10)	485 (63.56)	278 (36.44)
Suspicious malignancy *N*=26 (1.97%)	62.85 (17.90)	16 (61.54)	10 (38.46)
Specific benign results *N*=104 (7.89%)	57.62 (19.76)	45 (43.27)	59 (56.73)
Nonspecific benign results *N*=269 (20.43%)	60.72 (14.50)	146 (54.28)	123 (45.72)
Nondiagnostic results *N*=155 (11.77%)	59.67 (15.66)	69 (44.52)	86 (55.48)
Total	61.23 (15.04)	761 (57.78)	556 (42.22)

The average age of participants diagnosed with specific malignancy was 62.15 (SD=14.10) years. The most common specific malignant lesion was adenocarcinoma (*n*=328) (Table 2).

**Table 2:** The detailed pathology reports of specific malignant results of CT-PTNB

Pathology result	Frequency (%)
Adenocarcinoma	328 (42.98)
Non-small cell carcinoma	115 (15.07)
Poorly differentiated carcinoma	103 (13.49)
Squamous cell carcinoma	89 (11.66)
Spindle cell sarcoma	50 (6.55)
Small cell carcinoma	42 (5.51)
Lymphoma	14 (1.83)
Large cell carcinoma	7 (0.91)
Neuroendocrine carcinoma	5 (0.65)
Clear cell neoplasm	4 (0.52)
Plasma cell dyscrasia	4 (0.52)
Thymoma	2 (0.26)

The diagnostic outcome of CT-PTNB for pulmonary malignancy is presented in Table 3.

**Table 3:** Diagnostic outcome of CT-PTNB for pulmonary malignancy

Statistic	Value	95 % CI
Sensitivity	75.41	62.71–85.54
Specificity	91.43	76.94–98.20
Positive predictive value^a^	97.87	86.91–99.69
Negative predictive value^a^	68.09	57.79–76.88
Positive predictive value^b^	93.88	83.75–97.86
Negative predictive value^b^	71.11	60.11–80.08
Accuracy	82.98	73.84–89.95

a Nonevaluable results due to insufficient specimens were considered as false negatives.

b Nonevaluable results due to insufficient specimens were considered as false positives.

Moreover, adenocarcinoma was the most common final diagnosis in the specific malignancy (53.3%) and nonspecific benign results (17.8%) obtained by CT-PTNB. Among those identified as specific benign results by the CT-PTNB, hydatid cyst was found the most common final diagnosis (Fig. 1).

## Discussion

The present study measured the accuracy, sensitivity, and specificity of CT-PTNB for the diagnosis of pulmonary malignancy as 82.98, 75.41, and 91.43%, respectively.

The literature has consistently documented a high level of accuracy, ranging from 90 to 99%, in using CT-PTNB for the diagnosis of pulmonary malignancy^15–17^. However, our study demonstrated lower diagnostic accuracy as 82.98%; which could be attributed to diagnostic failures categorized into three types: false-positive, false-negative, and nonevaluable results^3,4^. Also, the sensitivity of CT-PTNB demonstrated in this study (75.41%) was lower compared to other studies^8,18–21^.

As per the guidelines outlined in the 2020 Clinical Practice Korean Guideline for Percutaneous Transthoracic Needle Biopsy of Pulmonary Lesions, it is recommended that the sensitivity of the biopsy for malignancy surpasses 85%, while the specificity should exceed 90%^6^.

Due to inadequate documentation, the exact reason of lower sensitivity and diagnostic accuracy is unclear. According to the previous studies, probable reasons could be considered as following: (i) differences in size, location, depth, and type of PPLs can effect on sensitivity and diagnostic accuracy^8,13,16,22–24^, and (ii) a disparity between the displayed location of the consumable tip on the screen and its actual location known as a skin shift^4,25^.

The incidence of specific malignancy lesions among 1317 cases of our study, reported to be 57.93%; which is notably lower than the median incidence rate of 69.4% reported in a recent meta-analysis^21^. This difference may be attributed to the lower sensitivity and accuracy of CT-PTNB in our study, resulting in the underdiagnosis of certain malignant cases. On the other hand, two-fifths of the studies included in this meta-analysis had an unclear risk of bias in terms of population selection, some of which encompassed high-risk populations, such as smokers.

The specificity of CT-PTNB in the diagnosis of pulmonary malignancies in this study was lower than the average specificity of previous studies^17,26–28^.

However, when an insufficient specimen (nonevaluable results) was regarded as a diagnostic failure, as was the case in our study, the combined sensitivity and specificity of PTNB procedures were ~90%^21^. In a study by Lee *et al*.^8^, which also utilized the intention-to-diagnose approach similar to our study, a specificity of 86.5% (lower than that of our study) was reported. Likewise, according to the 2020 Clinical Practice Korean Guideline^6^, it can be concluded that the specificity of our study is acceptable.

Recently, there has been a growing concern over nondiagnostic results obtained from PTNBs, a phenomenon that has introduced a significant clinical dilemma and ambiguity in decision-making. Several studies have reported varying rates of nondiagnostic lesions in PTNBs, ranging from 15 to 28%^24,29,30^. This variability in rates may be attributed to imaging modality and lesion size, with smaller lesions being associated with a higher incidence of nondiagnostic outcomes. In the present study, we observed a lower prevalence of nondiagnostic results (11.7%). This discrepancy could be attributed to utilization of CT-guided PTNB as the diagnostic modality of choice in our study, capable of accurately biopsying lesions greater than 1 centimeter in size. Conversely, a higher rate of nondiagnostic results in Lee *et al*.^24^ study (28%), could be potentially related to implementing fluoroscopy as one of their imaging modalities.

It is crucial to consider rare differential diagnosis during the evaluation of pulmonary nodular lesions in PTNB results. As demonstrated in our study, three cases among 1317 CT-PTNB results were diagnosed as COVID-19, a condition reported to occasionally present as multiple solid nodules, thereby potentially mimicking primary lung cancer or metastatic lesions, as described in the previous studies^31–33^. Furthermore, there were 12 cases of CT-PTNB reports as a hydatid cyst, highlighting the fact that the old forms of this infection are characterized by a structured and compact anomalous pulmonary nodule, which has the potential to imitate the radiological features of lung cancer or metastasis^34^.

The present study is subject to various limitations, mainly due to its multicentric design. The heterogeneity of the operators conducting the biopsies and pathologists may have affected the overall consistency and reliability of our findings. However, a standardized and categorized academic protocol was followed in performing imaging, sample collection, preparation, and pathological examination, leading to the minimization of structural errors and operator-related errors in this study. Furthermore, given the inadequate documentation of patient data, the present study was unable to provide details on patients’ demographic characteristics, medical history, imaging findings, and complications associated with the procedure. However, the large population, multicentric study and using ‘intention to diagnose approach’ in analyzing the results could be deemed as the strengths of this study.

The present study revealed that CT-PTNB exhibits an acceptable specificity but a lower level of sensitivity and diagnostic accuracy when compared to other studies. Given the comparatively lower reported diagnostic accuracy and sensitivity in relation to similar studies, the findings of this research can be suggested as a recommendation for physicians to exercise increased vigilance when monitoring patients whose pathology samples fall under the nonspecific or nondiagnostic category. Additionally, future studies should prioritize the collection and analysis of more comprehensive information pertaining to patients’ medical histories and the characteristics of pulmonary lesions, with a specific emphasis on their size. This approach will facilitate the exploration of potential factors that contribute to the observed decline in diagnostic accuracy.

## Ethical approval

The study protocol was reviewed and approved by the local institutional review board of Mashhad University of Medical Science by IRB number of IR.MUM.fm.REC.1395.304. The research adheres to the ethical standards outlined in the Declaration of Helsinki and its later amendments.

## Consent

The study protocol was approved by the local institutional review board of Mashhad University of Medical Science, which determined as minimal risk due to retrospective observational nature of the study and that the waiver of consent was justified. All data analyzed in this study were anonymized and handled with strict confidentiality to ensure participant privacy.

## Source of funding

Not applicable.

## Author contribution

R.B. and A.J.: designed the study; F.S. and A.Z.: collected the data; F.S.: analyzed data; A.Z.: interpreted data; P.F. and S.S.: wrote the manuscript. All authors read, revised, and approved the final manuscript.

## Conflicts of interest disclosure

The authors declares no conflicts of interest.

## Research registration unique identifying number (UIN)

Not applicable.

## Guarantor

Farzad Sharifnezhad.

## Data availability statement

Datasets analyzed during the study are available from the corresponding author upon reasonable request.

## Provenance and peer review

Not commissioned, externally peer-reviewed.
